# Alzheimer’s disease frequency peaks in the tenth decade and is lower afterwards

**DOI:** 10.1186/s40478-019-0752-0

**Published:** 2019-07-03

**Authors:** Jose M. Farfel, Lei Yu, Patricia A. Boyle, Sue Leurgans, Raj C. Shah, Julie A. Schneider, David A. Bennett

**Affiliations:** 1 Rush Alzheimer’s Disease Center, Rush University Medical Center, 1750 W. Harrison, Suite 1000, Chicago, IL 60612 USA; 2 Department of Pathology, Rush University Medical Center, Chicago, IL USA; 3 Department of Geriatrics, University of Sao Paulo Medical School, Sao Paulo, Brazil; 4 Department of Neurological Sciences, Rush University Medical Center, Chicago, IL USA; 5 Department of Behavioral Sciences, Rush Medical College, Chicago, IL USA; 6 Department of Family Medicine, Rush Medical College, Chicago, IL USA

**Keywords:** Alzheimer’s disease, Age, Neuropathology, Tau protein, Amyloid

## Abstract

Age is the most robust risk factor for Alzheimer’s dementia, however there is little data on the relation of age to Alzheimer’s disease (AD) and other common neuropathologies that contribute to Alzheimer’s dementia. We use data from two community-based, clinical-pathologic cohorts to examine the association of age with AD and other common pathologies. Participants were 1420 autopsied individuals from the Religious Orders Study or Rush Memory and Aging Project who underwent annual clinical evaluations for diagnosis of Alzheimer’s dementia, mild cognitive impairment (MCI), and level of cognition. The neuropathologic traits of interest were pathologic AD according to modified NIA-Reagan criteria, three quantitative measures of AD pathology (global AD pathology score, β-amyloid load and PHFtau tangle density), macro- and micro-scopic infarcts, neocortical Lewy bodies, TDP-43 and hippocampal sclerosis. Semiparametric generalized additive models examined the nonlinear relationship between age and the clinical and pathological outcomes. The probability of Alzheimer’s dementia at death increased with age such that for every additional year of age, the log odds of Alzheimer’s dementia was 0.067 higher, corresponding to an odds ratio of 1.070 (*p* < 0.001). Results were similar for cognitive impairment and level of cognition. By contrast, a nonlinear relationship of age with multiple indices of AD pathology was observed (all ps < 0.05), such that pathologic AD reached a peak around 95 years of age and leveled off afterwards; the quantitative measures of AD pathology were significantly lower at ages above 95. The association of age with other neuropathologies was quite distinct from that of AD in that most increased with advancing age. AD pathology appears to peak around 95 years of age while other common pathologies continue to increase with age.

## Introduction

Increasing age is the most robust risk factor for Alzheimer’s dementia. Population-based studies show that the age-specific incidence and prevalence of Alzheimer’s dementia increase markedly after age 65 [9, 19, 21]. Most studies suggest that the incidence and prevalence of Alzheimer’s dementia continues to increase across the entire age spectrum, including in the oldest-old [6, 7]. Although data from one study suggests that the incidence and prevalence may decline slightly after age 95 raising the possibility that the effect of age on Alzheimer’s dementia risk might attenuate in the 10th decade [23, 32].

The newly developed National Institute on Aging and Alzheimer’s Association (NIA-AA) framework defines Alzheimer’s disease (AD) pathologically, based on findings from neuroimaging or biofluid biomarkers, or autopsy [15]. While several studies suggest that the relation of AD to dementia is weaker in the oldest-old [11, 29], limited data is available on the association of age with AD and other common neuropathologies in very old persons [5, 26]. In a recent study, we found indirect evidence suggesting a non-linear relationship between age and AD pathology in a study examining sex differences in AD and other neuropathologies [27]. Here, we use data from two community-based cohort studies of aging in which all participants enroll without known dementia and are organ donors, the Religious Orders Study (ROS) and the Rush Memory and Aging Project (MAP). We extend our prior work by examining both linear and non-linear associations of age with Alzheimer’s dementia, cognition, multiple indices of AD and other common neuropathologies in more than 1400 persons aged 66 to 108 at death.

## Methods

### Participants

Data came from ROSMAP, a pair of community-based, clinical-pathologic cohort studies of aging and dementia. ROS enrolled older Catholic nuns, priests and brothers across the United States since 1994. MAP enrolled older residents in the Chicago metropolitan area since 1997. Participants signed an informed consent and an Anatomic Gift Act agreeing to post-mortem brain donation. Participants were free of known dementia at enrollment. Both studies were approved by the Institutional Review Board of Rush University Medical Center. Combined, the overall follow-up rate exceeds 95% and the autopsy rate exceeds 85%. Detailed information for ROSMAP is available (www.radc.rush.edu) [1].

At the time of these analyses, 1444 participants had undergone complete autopsy. We excluded 24 participants diagnosed with non-Alzheimer’s dementia. Of the remaining 1420, the average age at death was 89.0 years (SD: 6.7 years, range 65.9 to 108.3 years). There were 267 participants (18.8%) who died at the age 95 years and older, including 63 (4.4%) who died at the age of 100 years and older (Table 1).

**Table 1:** Demographical, clinical, and neuropathological characteristics of the sample by age at death

	<75Yr (*N* = 41)	75-79Yr (*N* = 90)	80-84Yr (*N* = 238)	85-89Yr (*N* = 398)	90-94Yr (*N* = 386)	95-99Yr (*N* = 204)	≥100Yr (*N* = 63)	Total (*N* = 1420)
Female sex, No. (%)	16 (39)	57 (64)	139 (58)	251 (63)	272 (70)	157 (77)	55 (87)	947 (67)
Education, mean (SD), years	18 (4)	16 (4)	17 (4)	16 (4)	16 (3)	16 (4)	16 (3)	16 (4)
AD Dementia No. (%)	5 (12)	19 (21)	86 (36)	169 (42)	191 (49)	112 (55)	35 (56)	617 (43)
Cognitive Impairment No. (%)	13 (32)	37 (41)	146 (61)	263 (66)	288 (75)	164 (80)	50 (79)	961 (68)
Global Cognition, mean (SD)	−0.3 (1.2)	−0.4 (1.0)	−0.7 (1.2)	−0.9 (1.2)	−1.1 (1.4)	−1.2 (1.1)	−1.4 (1.1)	− 0.9 (1.2)
Episodic Memory, mean (SD)	−0.2 (1.3)	−0.2 (1.2)	− 0.7 (1.3)	−0.9 (1.4)	− 1.1 (1.3)	− 1.3 (1.3)	− 1.3 (1.3)	− 0.9 (1.4)
AD Diagnosis No. (%)	6 (15)	39 (43)	132 (55)	262 (66)	275 (71)	155 (76)	48 (76)	917 (65)
Global AD Score, mean (SD)	0.4 (0.4)	0.6 (0.4)	0.7 (0.4)	0.8 (0.4)	0.8 (0.4)	0.8 (0.3)	0.8 (0.3)	0.8 (0.4)
Amyloid load, mean (SD)	0.8 (0.9)	1.1 (1.1)	1.3 (1.1)	1.7 (1.7)	1.8 (1.1)	1.8 (1.0)	1.7 (1.1)	1.6 (1.1)
PHF-tau tangles density, mean (SD)	0.8 (1.7)	1.0 (1.3)	1.4 (1.4)	1.7 (1.3)	1.7 (1.2)	1.7 (1.1)	1.7 (0.9)	1.6 (1.3)
Macroscopic infarcts, No. (%)	5 (12)	21 (23)	70 (29)	135 (34)	150 (39)	82 (40)	34 (54)	497 (35)
Microscopic infarcts, No. (%)	12 (29)	20 (22)	69 (29)	97 (24)	131 (34)	67 (33)	30 (48)	426 (30)
Cortical Lewy bodies, No. (%)	4 (10)	5 (6)	28 (12)	55 (14)	54 (14)	25 (13)	7 (11)	178 (13)
TDP-43 inclusions, No. (%)	1 (4)	7 (9)	35 (21)	110 (33)	129 (37)	89 (46)	29 (50)	400 (33)
Hippocampal sclerosis No. (%)	1 (3)	3 (3)	12 (5)	29 (7)	43 (11)	27 (13)	12 (19)	127 (9)
*APOE* ε4 carriers, No. (%)	9 (22)	25 (29)	82 (35)	113 (29)	92 (24)	44 (22)	8 (13)	373 (27)

AD denotes Alzheimer’s Disease

### Clinical assessment

Participants underwent uniform, annual clinical evaluations including a detailed cognitive assessment with a structured battery of 21 cognitive performance tests. Diagnostic classification of Alzheimer’s dementia proceeded in a multi-step process based on accepted criteria [22]. Mild cognitive impairment (MCI) referred to those persons with cognitive impairment without dementia. Of the 19 tests in common among the two cohorts, 17 were used to make a composite measure of global cognition, and seven were used to make a composite measure of episodic memory, as previously described [2].

### Neuropathological assessment

Brain removal and processing followed a standard protocol previously detailed [1]. Five regions were dissected and stained with modified Bielschowsky to assess for neuritic plaques (NP), diffuse plaques (DP), and neurofibrillary tangles (NFT). CERAD criteria was derived from NP counts and Braak stage was derived from the distribution and severity of NFTs [4, 24]. The three pathologic indices were also counted separately in each of the five regions in the area of the slide showing greatest density of pathology using a graticule to mark a 1-mm^2^ area at a magnification of 100X. The procedure resulted in 15 individual measures of pathology for each person that were averaged. to generate a global AD score, as previously described [3]. AD diagnosis was based on the modified National Institute on Aging (NIA)–Reagan criteria [13]. The diagnosis required an intermediate or high likelihood pathologic AD.

Immunohistochemistry and computer-assisted image analysis were used to estimate β-amyloid load across eight different brain regions. Immunohistochemistry and stereology was used to estimate Paired helical filament tau (PHFtau) tangle density and averaged across all regions as previously reported [18].

Lewy bodies (LB) were identified using alpha-synuclein immunostaining and recorded as absent, nigral, limbic, or neocortical [31]. Hematoxylin and eosin (H&E) was used to histologically confirm the presence of macroscopic infarcts, document microscopic infarcts in at least 9 regions, and to identify severe neuronal loss and gliosis in the hippocampus, characterized as hippocampal sclerosis (HS) [25, 28]. Cytoplasmic inclusions of TDP-43 pathology were assessed with immunostaining in 6 brain regions [25].

### *APOE* genotyping

DNA was extracted from white blood cells or frozen brain tissue. *APOE* genotyping was obtained by sequencing the codons 112 and 158 of exon 4 of the *APOE* gene. Participants with at least one copy of the ε4 were considered as ε4 carriers [8].

### Statistical analysis

First, we examined the association of age with Alzheimer’s dementia. We started with a logistic regression model with Alzheimer’s dementia as the binary outcome. This model examines the linear relationship between age and log odds (logit) of Alzheimer’s dementia. The corresponding regression coefficient estimates the difference in the logit of Alzheimer’s dementia with every 1 additional year of age. To assess the nonlinear relationship of age, we employed a semiparametric generalized additive model with a logit link function. In addition to a parametric linear term of age, this model also included a nonparametric cubic spline term for age [12]. The deviance between the models with and without the spline term was compared using a χ^2^ test with 3 degrees of freedom. A significant test statistic indicates that the spline term improves the model fit, thus supporting a nonlinear relationship of age with the logit. The presence of a peak age for Alzheimer’s dementia was determined by plotting the model predicted probabilities of Alzheimer’s dementia against age. We repeated the analysis for cognitive impairment (combination of MCI and Alzheimer’s dementia). Separately, we repeated the analysis to examine the association of age with the diagnosis of pathologic AD according to NIA-Reagan criteria. We repeated the analysis by adjusting for *APOE* ε4 status.

Linear regression models examined the linear association of age with quantitative measures of global cognition, and episodic memory, the clinical hallmark of AD, proximate to death, and the three quantitative AD pathologic indices (i.e. global AD score, β-amyloid load and PHFtau tangle density, all square root transformed due to positive skewness. In these models, each cognitive or AD measure was the continuous outcome and age was the predictor. The regression coefficient for age estimates the difference in cognition or the burden of AD with every additional year of age. Semiparametric generalized additive models with an identity link function examined the nonlinear relationship of age. Similar to the analysis for AD dementia, these additive models included both a linear term and a cubic spline term for age. Without the spline term, the models reduce to regular linear regression models. A χ^2^ statistic compared the deviance between the models with and without the spline term, and a significant test statistic supports a nonlinear relationship of age with the quantitative measure of cognition or AD. We repeated the analyses by adjusting for *APOE* ε4 status.

Finally, we repeated the analyses for the presence or absence of non-AD pathologies including HS, cerebral infarctions, cortical LB, and TDP43 pathology. In secondary analyses, we excluded 12 individuals aged 103 years and above to ensure that the results were not driven by extremely old subjects. All models were adjusted for sex and education, and the analyses were performed using SAS/STAT software, version 9.4 [SAS Institute Inc., Cary, NC]. Statistical significance was determined at the nominal level of *p* < 0.05.

## Results

Demographic, clinical, and neuropathological characteristics by age groups at death, not adjusted for covariates, are shown in Table 1. The age-specific estimated probability of Alzheimer’s dementia increased with age with the highest probability (56%) for the oldest-old (≥100 years). Findings were similar for the continuous measures of global cognition and episodic memory which decreased with age. By contrast, pathologic AD reached a peak of estimated probability (76%) in the age group 95 to 100 following which it did not increase. Findings were similar for the continuous measures of global AD score, β-amyloid load, and PHFtau tangle density. Macro and microscopic infarctions had the highest probability over age 100, as did TDP-43 and HS. By contrast, the frequency of cortical LB remained stable across the age spectrum.

**Fig. 1:**
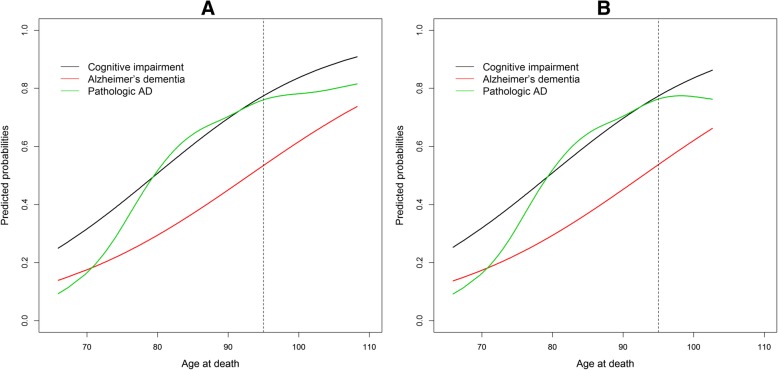
Estimated probabilities of Alzheimer’s dementia, cognitive impairment and AD TRUN 0TQCLICKWFI 1diagnosis according to age. Data are from separate models shown superimposed for comparison. Panel **a** includes all participants whereas Panel **b** excludes participants aged 103 or older

We first examined the association of age with Alzheimer’s dementia, adjusted for sex and education. We found a linear association of age with the logit. For each additional year of age, the logit was 0.067 higher, corresponding to an OR of 1.070 (95% Confidence Interval [CI]: 1.051–1.088, *p* < 0.001). Thus, the odds of Alzheimer’s dementia nearly doubles for each decade of life. The inclusion of a spline term for age to examine non-linearity in the model did not improve the fit (*p* = 0.15). The estimated probability of Alzheimer’s dementia continues to rise with age from about 15% at age 70, to about 40% at age 90, to about 65% at age 105 (Fig. 1a, red line). Separately, we found a linear association of age with the logit of cognitive impairment. With each additional year of age, the logit of having cognitive impairment was 0.080 higher, corresponding to an OR of 1.083 (95% CI, 1.064–1.103, *p* < 0.001). The inclusion of a spline term for age did not improve the model fit (*p* = 0.39). The estimated probability of cognitive impairment increased with age from about 30% at age 70, to about 65% at age 90, to nearly 90% at age 105 (Fig. 1a, black line).

Next, we examined the association of age with pathologic AD. With each additional year of age, the logit of having AD was 0.075 higher (OR = 1.078, 95% CI: 1.059–1.097, *p* < 0.001), nearly doubling for each decade. In contrast to the findings for Alzheimer’s dementia and cognitive impairment, we observed an improvement in the model fit after including a spline term for age to examine non-linearity (χ^2^(3) = 11.05, *p* = 0.01). The estimated probability for AD diagnosis increased rapidly from about 15% at age 70 to about 65% at age 85, it was slightly higher at about 75% by age 95, following which it was essentially flat (Fig. 1a, green line). To ensure that the nonlinear relationship was not driven by extremely old subjects, we reanalyzed the data after excluding participants aged 103 and above. The findings were similar, but the downturn after age 95 was more pronounced (Fig. 1b, green line). To examine if the findings were affected by *APOE* we repeated the analysis controlling for *APOE* ε4 status and the overall findings were similar (data not shown).

**Fig. 2:**
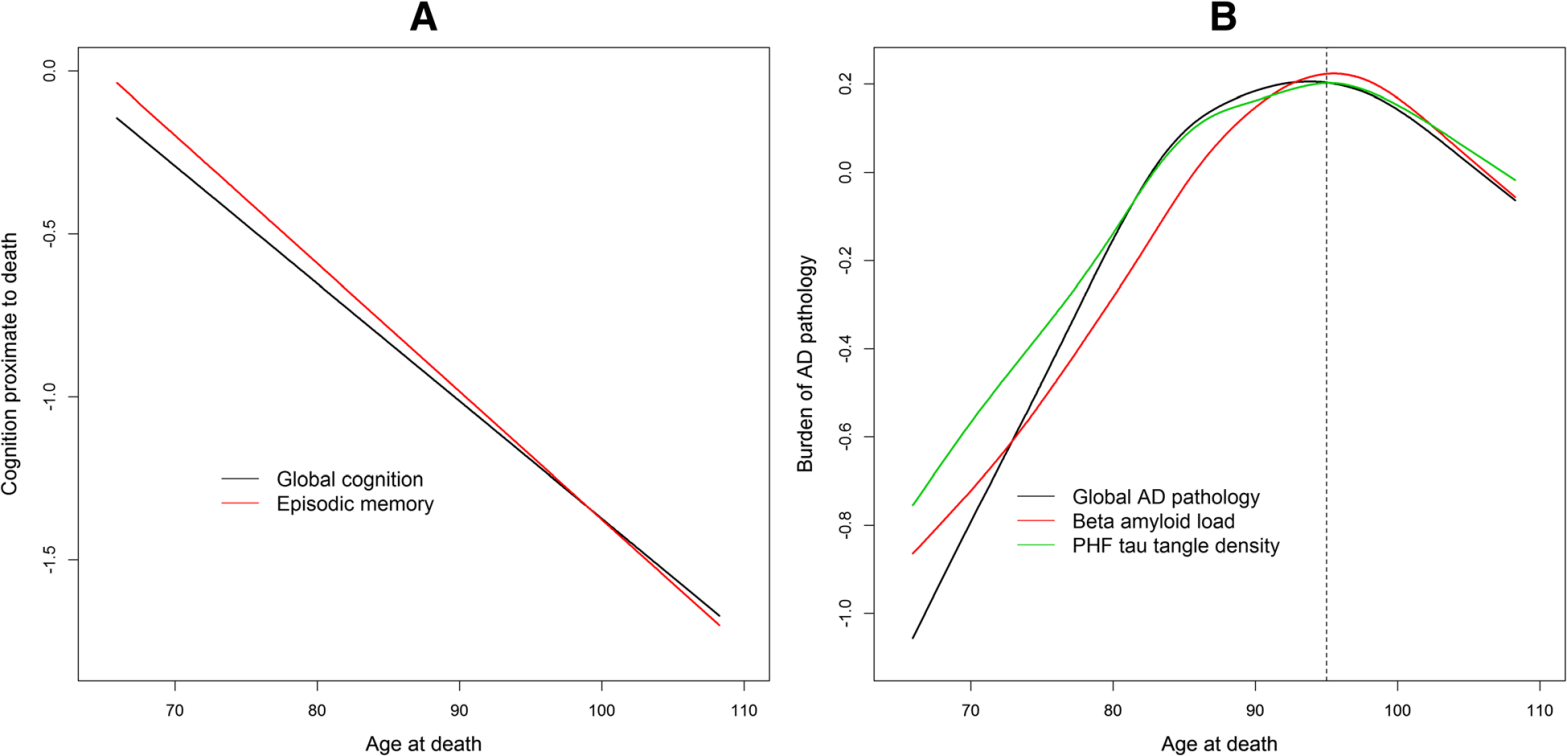
Associations of age with quantitative measures of cognition and Alzheimer’s disease. Panel **a** shows association of age with cognition proximate to death; Panel **b** shows association of age with quantitative pathologic measures of Alzheimer´s disease. Data are from separate models shown superimposed for comparison

Because Alzheimer’s dementia, cognitive impairment, and AD diagnosis each represent a dichotomization on an underlying continuum [15], we next examined the association of age with continuous measures of cognition and AD pathology. We first examined the association of age with the level of global cognition and episodic memory, the clinical hallmark of Alzheimer’s dementia, proximate to death. Similar to the findings for Alzheimer’s dementia and cognitive impairment, we found strong linear associations of both global cognition (*p* < 0.001) and episodic memory (*p* < 0.001), with no evidence of a nonlinear association of age with global cognition (*p* = 0.45) or episodic memory (*p* = 0.39) (Fig. 2a, black and red, respectively). We next examined the associations of age with three different quantitative measures of AD pathology. In contrast to level of cognition, we observed highly significant nonlinear associations of age with global AD pathology score for which the evidence was much stronger than the association with binary AD diagnosis (χ^2^(3) = 23.6, *p* < 0.001), β-amyloid load (χ^2^(3) = 15.9, *p* = 0.001), and PHFtau tangle density (χ^2^(3) = 13.4, *p* = 0.004) (Fig. 2b, black, red, and green, respectively). For all three outcomes it is evident that the model predicted values leveled off after the age of 85 years, and after the age of 95 they became significantly lower. The findings were unchanged after excluding super-agers or after controlling for *APOE* ε4 status (data not shown).

**Fig. 3:**
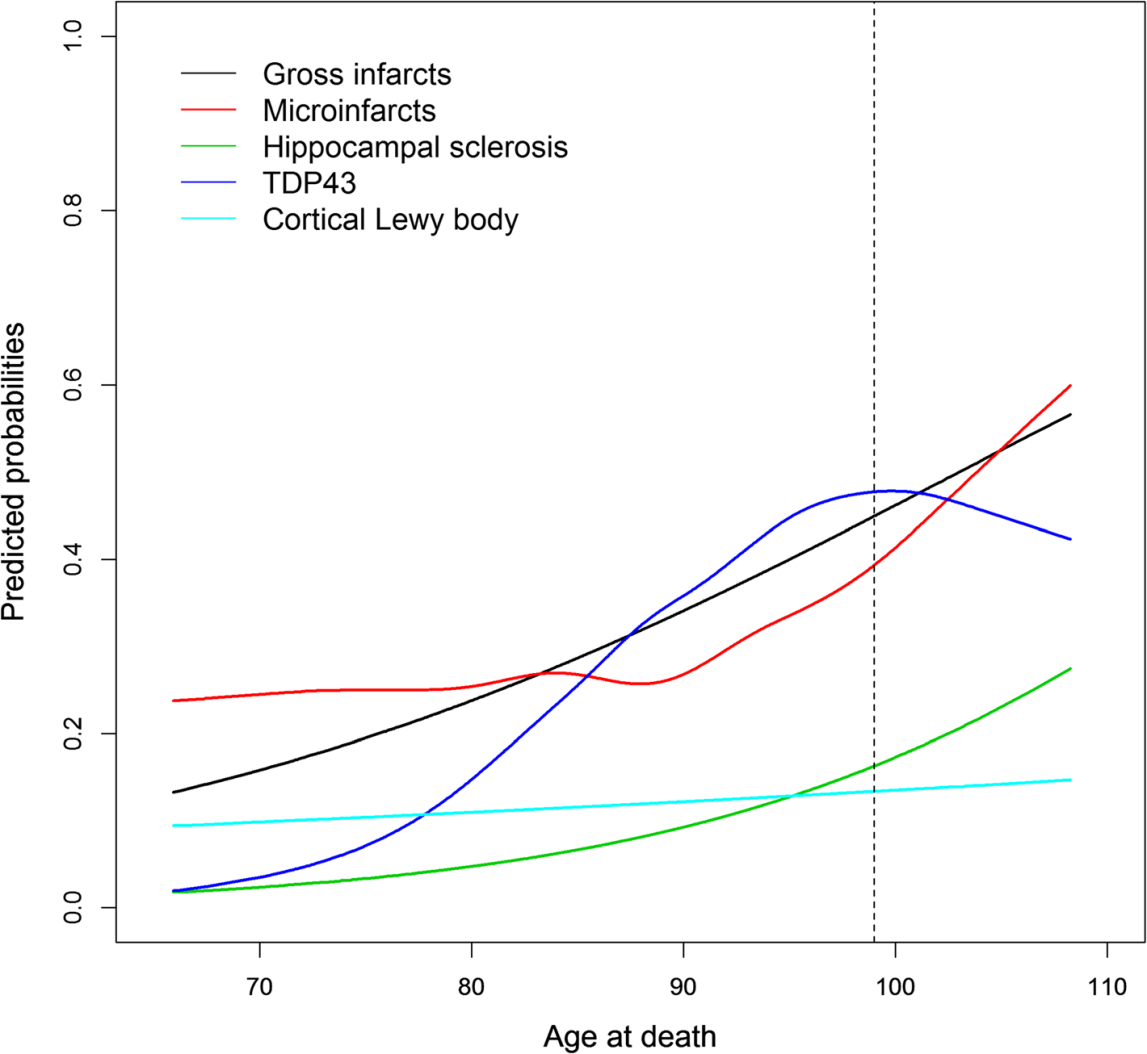
Estimated probabilities of non-AD neuropathologies according to age. Data are from separate models shown superimposed for ease of visual comparisons

Given that the estimated probabilities of Alzheimer’s dementia and cognitive impairment increased past age 95 and AD pathology leveled off and became lower after age 95, we anticipated that other pathologies would continue to increase with age. In the final set of analyses, we examined the association of age with five non-AD neuropathologies. Older age was associated with greater odds of macroinfarcts (OR = 1.052, 95% CI = 1.033–1.071, *p* < 0.001) and HS (OR = 1.074, 95% CI = 1.042–1.108, *p* < 0.001). For these two pathologies, the associations were driven by the linear term of age with the logit, whereas the spline term was not significant (p’s = 0.10 and 0.63- respectively) (Fig. 3, black and green). We found nonlinear associations of age with the logits of microinfarcts (χ^2^(3) = 9.9, *p* = 0.02) and TDP-43 (χ^2^(3) = 9.4, *p* = 0.02). Notably, however, one of these nonlinear relationships differed from those for AD pathology. Specifically, the estimated probability of microinfarcts was relatively stable until approximately 90 years, after which there was a sharp increase with age (Fig. 3, red). Separately, the probability of having TDP-43 pathology increased until around age 98, after which a downward trend was observed (Fig. 3, purple). Unexpectedly, we did not observe an association of age with neocortical LB (*p* = 0.34). The estimated probability of neocortical LB is approximately 10% and remained stable across the age spectrum (Fig. 3, light blue). After excluding the super-agers, the nonlinear term was no longer significant for TDP-43 pathology (*p* = 0.15), and the findings for other pathologies were unchanged (data not shown). Finally, we repeated the analysis by adding the 24 subjects with the diagnosis of non-AD dementia and the results were unchanged.

## Discussion

Multiple indices of Alzheimer’s dementia, cognitive impairment, cognitive function, AD and other common neuropathologies were assessed in two community-based cohort studies with more than 1400 older persons covering a range of age at death of more than four decades. We found that the probability of Alzheimer’s dementia and cognitive impairment proximate to death increased with advancing age; correspondingly, level of cognition decreased with advancing age. By contrast, pathologic AD showed a peak probability at death around the age of 95 years and following which it did not increase further. The findings were much more striking with three different quantitative measures of AD pathology all of which were significantly lower as age increased past 95 years. The findings were robust and not driven by small numbers at extreme old age or *APOE* status*.* By contrast, four other non-AD neuropathologies increased dramatically with age after 95.

Little data from other studies focusing in other non-AD pathologies are available on this topic [5, 26]. One prior study on hippocampal sclerosis found that AD peaked at about age 95 after which it declined, while hippocampal sclerosis increased well past age 100 [26]. In that case-control study, the decrease found for AD was not formally tested for significance. Participants were mostly from a tertiary care clinic with additional samples from the Nun Study and a centenarian study. Clinic cases differ from community-dwelling elderly, centenarian studies are left censored, most participants had severe cognitive impairment, and case-control designs are susceptible to bias raising questions about the veracity of the finding. We designed our study to confirm and extend that finding in several ways. We contextualized the pathology contrasting it with clinical observations from the same persons covering the full spectrum of cognition. Further, we formally tested non-linearity for significance. We also illustrated the robustness with quantitative measures of AD pathology, and by sensitivity analyses dropping those at extreme age and adjusting for *APOE*. Finally, we examined five additional common neuropathologies and found strikingly different associations with age.

The explanation of age as a risk factor for AD has been the subject of intense investigation for many years. Most hypotheses consider age a metric of time on Earth such that continuous dysfunctional processes lead to pathology deposition and eventual cognitive impairment. For example, an imbalance between β-amyloid production and clearance could trigger a cascade of events resulting in deposition of β-amyloid, tangle formation, and cognitive decline [10]. Genetic and other time-invariant risk factors may affect this imbalance and the timing of the events of the cascade resulting in their association with age [30]. It is likely that subgroups at high risk developed and died from Alzheimer’s dementia by the tenth decade and exited the population. This differential survival can result in an exhaustion of known and unknown genetic or other time-invariant risk factors and in a lower frequency of AD consistent with the findings of our study. It is noteworthy that we did not find similar effect for several other pathologies suggesting that AD is relatively unique in that regard. Thus, our finding is a demonstration that AD reaches a peak age after which it is censored by death with AD. We adjusted our models for *APOE* and our finding for AD were unchanged. The differential survival is probably related to other genetic or time-invariant risk factors, not entered in our models. Future investigations are needed to determine the risk factors related to AD differential survival.

While AD reached a peak at about age 95, Alzheimer’s dementia, cognitive impairment, and level of cognition all continued to worsen into the eleventh decade. Therefore, we also examined the association of age with five common non-AD pathologies. In contrast to measures of AD, the estimated probability of macroscopic infarctions, and hippocampal sclerosis at death continued to increase with age. The probability of microscopic infarctions at death also increased with age with the effect opposite that of AD in that it increased more rapidly after age 90. Further, TDP-43 likewise increased with age. Interestingly, age was not related to neocortical LB. Our data is consistent with prior studies showing a steady rise of non-AD pathologies with advancing age [5, 14, 26] and with others that found that pathologies other than AD become increasingly more important drivers of dementia and cognitive impairment in the oldest-old [17, 20]. These findings suggest that interventions that target non-AD neuropathologies, specifically hippocampal sclerosis, TDP-43 pathology, and vascular disease may be of greatest utility in the oldest-old, the most rapidly growing segment of the population in the USA and other developed countries.

This study has a number of strengths. The cohort studies enroll persons without dementia and have very high rates of clinical follow-up and autopsy, which increases the internal validity of findings. The study includes large numbers of persons with and without cognitive impairment and AD at very old ages. We examined multiple clinical measures and multiple features of AD, and the results were highly consistent and robust. These strengths lend confidence to the findings. The study also has limitations. Age as a metric provides the frequency of prevalent but not incident pathologies at death. While we had excellent coverage of ages up to about 105, it is possible that other pathologic indices also show an age peak at more extreme ages. The cohorts enroll persons on average ages 75 and 80, without dementia, leading to a healthy volunteer effect to some degree. We strongly considered the possibility of survival bias as discussed above. However, but this is highly unlikely given the strikingly different patterns seen for AD and for both the clinical findings as well as all of the other non-AD pathologies. It would be very peculiar for survival bias to be specific for AD pathologies. Thus, while the findings are likely due to differential survival, this difference does not bias our findings but rather explains it. Ultimately, biofluid and neuroimaging biomarker data will be needed to complement our findings in living persons. Unfortunately, such data is not currently available in persons over age 90, let alone the large numbers who reach the eleventh decade of life needed for robust non-linear modeling [16].

## Conclusion

In this manuscript, we found that AD pathology, measured through four different phenotypes, reaches a peak around 95 years of age and is lower afterwards. By contrast, the frequency of Alzheimer’s dementia, cognitive impairment, and impaired cognition, as well as measures from four other non-AD pathologies continue to increase with age. The findings of this study may influence basic biological concepts in the field and highlight the public health need to further understand the biology of AD and other common neuropathologies in the rapidly growing demographic 90+ age group. Further, the findings suggest that interventions that target neuropathologies other than AD, specifically hippocampal sclerosis, TDP-43 pathology, and vascular disease may be of greatest utility in the oldest old.

## Data Availability

Data from this study can be requested at the RADC Resource Sharing Hub (www.radc.rush.edu).

## Abbreviations

AD: Alzheimer’s disease
DP: Diffuse plaques
H&E: Hematoxylin and eosin
HS: Hippocampal sclerosis
LB: Lewy bodies
MAP: Rush Memory and Aging Project
MCI: Mild cognitive impairment
NFT: Neurofibrillary tangles
NIA-AA: National Institute on Aging and Alzheimer’s Association
NP: Neuritic plaques
PHFtau: Paired helical filament tau
ROS: Religious Orders Study
